# Letter from A. W. French

**Published:** 1854-01

**Authors:** A. W. French


Springfield, Ill., *Nov. 22d* 1853-

*Editor of the Dental Register :*

Dear Sir: I regret that my letter to you, was so badly written that it could not be read correctly; and I take the liberty of sending some corrections, which you can dispose of as you think best.

[The communication of Dr. French was written plainly enough, and we cannot now say, whether these errors are typographical or not.—Ed.]

On the 55th page, a sentence amended, should read, "Every dentist knows very well, that it is one thing to fashion and finish a plate" &c.

Lower down on the same page, for "performing perfectly for gastric elaborations," read preparing perfectly, &c.

Lower still, on the same page, for "sham," read show.

56th page. The last word of the paragraph, viz., "incorrect," should be inconvenient. Also, my initials.

Your obedient servant, A. W. FRENCH.

